# Supplementary material for: Polyaniline-Coated Porous Vanadium Nitride Microrods for Enhanced Performance of a Lithium–Sulfur Battery
Source: Molecules. 2023 Feb 15;28(4):1823. doi: 10.3390/molecules28041823 (PMC9967358; doi:10.3390/molecules28041823)
Supplement: Supplementary file 1 [file molecules-28-01823-s001.zip › molecules-2168037-supplementary.pdf]

Supporting information

Polyaniline-Coated Porous Vanadium Nitride Microrods for Enhanced Performance of a Lithium–Sulfur Battery

Jingjie Lv [1], Haibo Ren [2,*], Ziyan Cheng [1], Sang Woo Joo [3,*] and Jiarui Huang [1,*]

[1] Key Laboratory of Functional Molecular Solids of the Ministry of Education, Anhui Laboratory of Molecule-Based Materials, College of Chemistry and Materials Science, Anhui Normal University, Wuhu 241002, China
[2] School of Materials Science and Engineering, Modern Technology Center, Anhui Polytechnic University, Wuhu 241000, China
[3] School of Mechanical Engineering, Yeungnam University, Gyeongsan 712749, Republic of Korea
* Correspondence: renhaibo@ahpu.edu.cn (H.R.); swjoo@yu.ac.kr (S.W.J.); jrhuang@ahnu.edu.cn (J.H.)

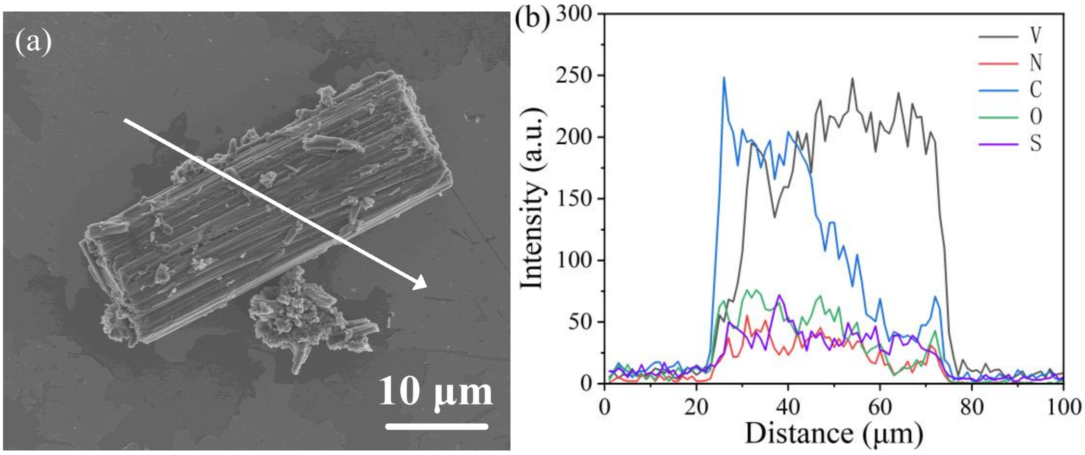

Figure S1. (a) SEM image of VN/S@PANI composite. (b) Line scanning curves of VN/S@PANI composite.

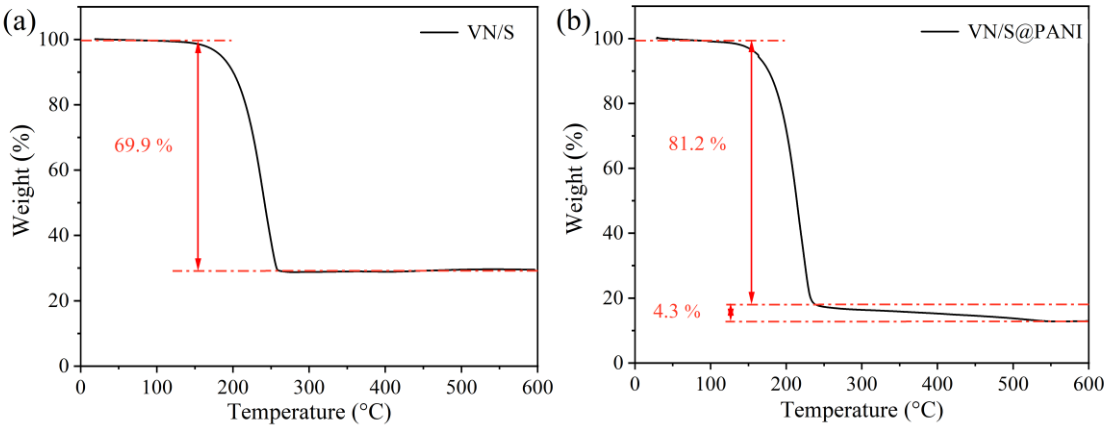

Figure S2. TGA curves of (a) VN/S microrods and (b) VN/S@PANI composite.

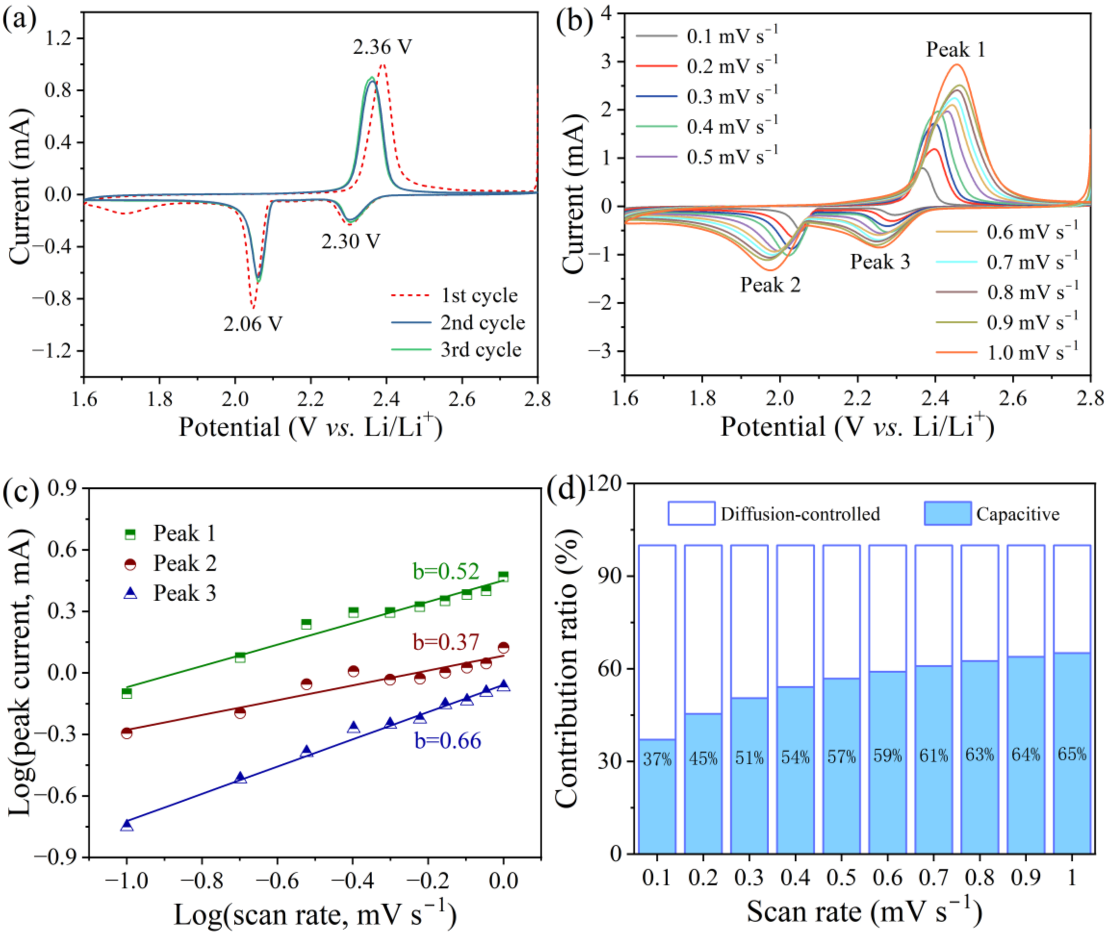

Figure S3. (a) Initial five CV curves of VN/S microrod cathode at a scan rate of 0.1 mV s^{-1}. (b) CV curves of VN/S microrod cathode at 0.1 to 1.0 mV s^{-1}. (c) The log(i) *vs.* log(v) of VN/S microrod cathode. (d) Contribution ratio of capacitance control and diffusion control.

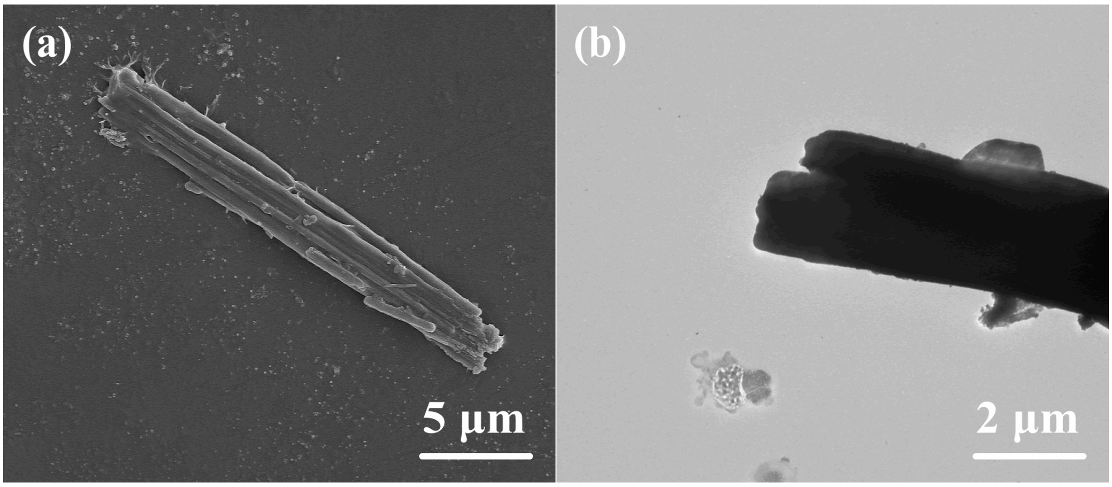

Figure S4. (a) SEM and (b) TEM images of VN/S@PANI microrod cathode after 500 cycles.

Table 1 Compared electrochemical performance of reported materials as sulfur hosts or interlayers.

Materials	Preparation method	Cycling rate (A g^{-1}) /Cycle number	Capacity (mAh g^{-1})	Ref.
VN/S microflowers	Solution-calcining method	5/500	62	[11]
PANI@BDC/S	Solution-calcining method	0.8/500	366	[35]
S/VN@CNFs	Electrospinning and spraying method	0.33/100	855	[38]
VN-NCNFs/S nanofibers	Electrospinning and spraying method	1.67/500	560	[39]
V_2O_3-VN@NC/S	Solution-calcining method	0.33/150	716	[40]
3DNPC/VN-S nanoparticles	Template method	1.67/300	615	[41]
CFP@PANI-PP	Hydrolysis method	1.67/200	583	[42]
V_2O_3/S	Hydrothermal method	0.33/200	509	[43]
AC@V_2O_5/S	Solution-calcining method	0.17/100	795	[44]
VN/S@PANI	Calcining method and coating	0.5/150<br>2.0/400	735<br>458	This work
